# RA-MAP, molecular immunological landscapes in early rheumatoid arthritis and healthy vaccine recipients

**DOI:** 10.1038/s41597-022-01264-y

**Published:** 2022-05-09

**Authors:** The RA-MAP Consortium, John D. Isaacs, Sarah Brockbank, Ayako Wakatsuki Pedersen, Catharien Hilkens, Amy Anderson, Philip Stocks, Dennis Lendrem, Jessica Tarn, Graham R. Smith, Ben Allen, John Casement, Julie Diboll, Rachel Harry, Faye A. H. Cooles, Andrew P. Cope, Gemma Simpson, Ruth Toward, Hayley Noble, Angela Parke, Wing Wu, Fiona Clarke, David Scott, Ian C. Scott, James Galloway, Heidi Lempp, Fowzia Ibrahim, Samana Schwank, Gemma Molyneux, Tomi Lazarov, Frederic Geissmann, Carl S. Goodyear, Iain B. McInnes, Iona Donnelly, Ashley Gilmour, Aysin Tulunay Virlan, Duncan Porter, Frederique Ponchel, Paul Emery, Jehan El-Jawhari, Rekha Parmar, Michael F. McDermott, Benjamin A. Fisher, Steve P. Young, Philip Jones, Karim Raza, Andrew Filer, Costantino Pitzalis, Michael R. Barnes, David S. Watson, Rafael Henkin, Georgina Thorborn, Liliane Fossati-Jimack, Stephen Kelly, Frances Humby, Michele Bombardieri, Sharmila Rana, Zhilong Jia, Katriona Goldmann, Myles Lewis, Sandra Ng, Adriano Barbosa-Silva, Evan Tzanis, Amaya Gallagher-Syed, Christopher R. John, Michael R. Ehrenstein, Gioia Altobelli, Sandra Martins, Dao Nguyen, Humayara Ali, Coziana Ciurtin, Maya Buch, Deborah Symmons, Jane Worthington, Ian N. Bruce, Jamie C. Sergeant, Suzanne M. M. Verstappen, Fiona Stirling, Adwoa Hughes-Morley, Brian Tom, Vernon Farewell, Yujie Zhong, Peter C. Taylor, Christopher D. Buckley, Sarah Keidel, Carolyn Cuff, Marc Levesque, Andrew Long, Zheng Liu, Samantha Lipsky, Bohdan Harvey, Michael Macoritto, Feng Hong, Sukru Kaymakcalan, Wayne Tsuji, Tony Sabin, Neil Ward, Susan Talbot, Desmond Padhji, Matthew Sleeman, Donna Finch, Athula Herath, Catharina Lindholm, Martin Jenkins, Meilien Ho, Sally Hollis, Chris Marshall, Gerry Parker, Matt Page, Hannah Edwards, Alexandru Cuza, Neil Gozzard, Ioannis Pandis, Anthony Rowe, Francisco Bonachela Capdevila, Matthew J. Loza, Mark Curran, Denny Verbeeck, Dan Baker, Christopher M. Mela, Ivana Vranic, Catherine T. Mela, Stephen Wright, Lucy Rowell, Emma Vernon, Nina Joseph, Neil Payne, Ravi Rao, Michael Binks, Alexandra Belson, Valerie Ludbrook, Kirsty Hicks, Hannah Tipney, Joanne Ellis, Samiul Hasan, Arnaud Didierlaurent, Wivine Burny, Andrea Haynes, Chris Larminie, Ray Harris, Daniela Dastros-Pitei, Claudio Carini, Blerina Kola, Scott Jelinsky, Martin Hodge, Mateusz Maciejewski, Daniel Ziemek, Peter Schulz-Knappe, Hans-Dieter Zucht, Petra Budde, Mark Coles, James A. Butler, Simon Read

**Affiliations:** 1 Translational & Clinical Research Institute, Faculty of Medical Sciences, Newcastle University, UK; 2 Musculoskeletal Unit, Newcastle upon Tyne Hospitals NHS Foundation Trust, Newcastle upon Tyne, UK; 3 Bioinformatics Support Unit, Faculty of Medical Sciences, Newcastle University, Newcastle Upon Tyne, UK; 4 Academic Department of Rheumatology, Division of Immunology, Infection and Inflammatory Disease, Faculty of Life Sciences, King’s College London, London, UK; 5 Department of Immunobiology, Division of Immunology, Infection and Inflammatory Disease, Faculty of Life Sciences, King’s College London, London, UK; 6 Institute of Infection, Immunity and Inflammation, College of Medical, Veterinary and Life Sciences, University of Glasgow, Glasgow, UK; 7 Gartnavel General Hospital, Glasgow, UK; 8 Leeds Institute of Rheumatic and Musculoskeletal Medicine (LIRMM), School of Medicine, and the Leeds NIHR Biomedical Research Centre, The University of Leeds, Leeds, UK; 9 University of Birmingham and University Hospitals Birmingham NHS Trust and Sandwell and West Birmingham Hospitals NHS Trust, Birmingham, UK; 10 University of Birmingham and Sandwell and West Birmingham Hospitals NHS Trust, Birmingham, UK; 11 Centre for Experimental Medicine and Rheumatology, William Harvey Research Institute, Faculty of Medicine and Dentistry, Queen Mary University of London, London, UK; 12 Centre for Translational Bioinformatics, William Harvey Research Institute, Faculty of Medicine and Dentistry, Queen Mary University of London, London, UK; 13 Division of Medicine, University College London, London, UK; 14 Centre for Musculoskeletal Research, NIHR Manchester Biomedical Research Centre, School of Biological Sciences, Faculty of Biology, Medicine and Health, University of Manchester, Manchester, UK; 15 MRC Biostatistics Unit, University of Cambridge, Cambridge, UK; 16 The Kennedy Institute of Rheumatology, University of Oxford, Oxford, UK; 17 Medical Affairs, Abbvie Ltd, Maidenhead, SL6 4UB UK; 18 Translational Immunology, AbbVie Bioresearch Center Inc, Worcester, MA USA; 19 Immunology Clinical Development, Abbvie Bioresearch Center Inc, Worcester, MA USA; 20 Immunology Pharmacology, AbbVie Bioresearch Center Inc, Worcester, MA USA; 21 Information Research, AbbVie Bioresearch Center Inc, Worcester, MA USA; 22 Global Biologics, AbbVie Bioresearch Center Inc, Worcester, MA USA; 23 Exploratory Statistics, AbbVie Bioresearch Center Inc, Worcester, MA USA; 24 Department of Neuroscience, Amgen, Inc, Thousand Oaks, CA 91320 USA; 25 Respiratory, Inflammation & Autoimmunity, MedImmune Ltd, Cambridge, UK; 26 Global Medicines Development, Astrazeneca, Cambridge, UK; 27 UCB Celltech, Slough, UK; 28 Janssen Research & Development Ltd, High Wycombe, UK; 29 Janssen Research & Development, LLC, Spring House, Pennsylvania, USA; 30 Janssen Pharmaceutica NV, 2340 Beerse, Belgium; 31 Roche Products Ltd. 6 Falcon Way, Shire Park, Welwyn Garden City, AL7 1TW UK; 32 GlaxoSmithKline R&D Stevenage, Stevenage, UK; 33 European Knowledge Centre, Eisai Ltd, Hatfield, AL10 9SN UK; 34 Inflammation and Immunology Research Unit, Worldwide Research & Development Pfizer Inc, Washington, USA; 35 International Developed Markets, Pfizer Ltd, Tadworth, Surrey, KT20 7NS UK; 36 Protagen AG, 44227 Dortmund, Otto-Hahn Street 15, Germany; 37 SimOmics Ltd, Kennedy Institute of Rheumatology, University of Oxford, Roosevelt Drive, Headington, Oxford, OX3 7FY UK; 38 Grunenthal GmbH, Zieglerstraße 6, 52078 Aachen, Germany

**Keywords:** Rheumatoid arthritis, Predictive markers, Genetics research, Diagnostic markers

## Abstract

Rheumatoid arthritis (RA) is a chronic inflammatory disorder with poorly defined aetiology characterised by synovial inflammation with variable disease severity and drug responsiveness. To investigate the peripheral blood immune cell landscape of early, drug naive RA, we performed comprehensive clinical and molecular profiling of 267 RA patients and 52 healthy vaccine recipients for up to 18 months to establish a high quality sample biobank including plasma, serum, peripheral blood cells, urine, genomic DNA, RNA from whole blood, lymphocyte and monocyte subsets. We have performed extensive multi-omic immune phenotyping, including genomic, metabolomic, proteomic, transcriptomic and autoantibody profiling. We anticipate that these detailed clinical and molecular data will serve as a fundamental resource offering insights into immune-mediated disease pathogenesis, progression and therapeutic response, ultimately contributing to the development and application of targeted therapies for RA.

## Abstract (summary)

**Table:** 

Measurement(s)	Transcriptomics, small RNA, proteomics, autoantibodies, metabolomics, genotype, blood, urine
Technology Type(s)	microarray, RNA sequencing, somascan, NMR metabolomics, luminex autoantibody array, exome array
Sample Characteristic - Organism	Homo sapiens

## Background & Summary

Rheumatoid arthritis (RA) is an immune-mediated inflammatory disease (IMID) that clinically manifests in the joints, but is systemic in impact. Early and intensive treatment is a critical determinant of long-term outcome, although clinical remission remains a minority outcome and sustained drug-free remission remains rare^1^.

**Fig. 1:**
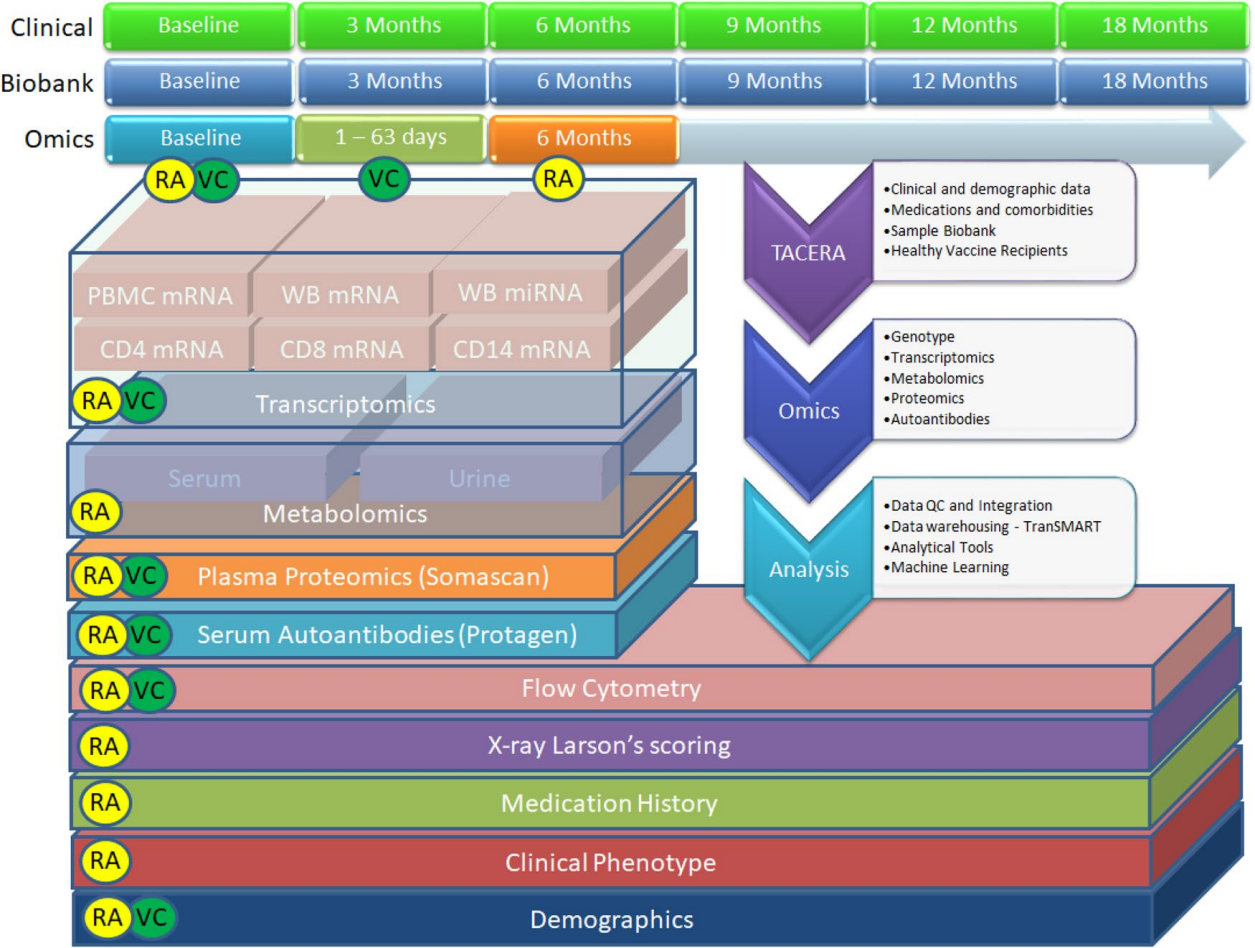
Overview of the RA-MAP project. a multi-omic bio-resource to facilitate the study of immune response in rheumatoid arthritis (RA) patients and healthy vaccine recipients (VC).

The RA-MAP Consortium is a UK industry-academic precision medicine partnership funded by the Medical Research Council and the Association of the British Pharmaceutical Industry (ABPI). RA-MAP’s goals are to investigate clinical and biological predictors of disease outcome and treatment response in RA, using deep clinical and multi-omic phenotyping (Fig. 1). The study is in part motivated to inform the study design and analysis of future studies of blood and immune cell subsets in RA and other IMIDs. RA-MAP patients follow the UK-NHS standard of care, with first-line treatment with conventional synthetic disease-modifying anti-rheumatic drugs (csDMARD), such as methotrexate, which have slow onset of action. In the case of non-responders to csDMARDs, prolonged periods of uncontrolled disease activity can lead to joint damage and disability. Thus, RA-MAP seeks to address a major unmet need to identify patient-level predictors of response in order to identify patients with a greater or lesser chance of clinically responding to csDMARD treatment. Such information could guide treatment choices, possibly supporting fast-track biologic therapy, leading to improved long-term outcomes for patients, and saving time and money in achieving sustained disease control and improving the efficiency of clinical trials.

Molecular profiling of whole blood and peripheral blood mononuclear cells (PBMCs) has been widely used to investigate the molecular heterogeneity and pathogenesis of RA across a number of transcriptome analysis platforms^2–8^. However only a small number of studies have profiled more than 100 patients^9–11^. Most studies have focused on patients with well-established disease who were already on DMARD therapy. Although findings of interest have been identified in these studies, such as association between therapeutic response and type I interferon signatures^8^, there remains a major unmet need for better molecular characterization of both heterogeneity and disease activity in RA without the confounding effects of immunomodulatory therapy.

The “Towards A Cure for Early Rheumatoid Arthritis” (TACERA) study^12^ was designed to detect distinct disease subtypes and the immunological correlates of disease activity and autoimmunity in patients with early, seropositive RA, with a secondary objective to investigate biomarkers of initial drug response to methotrexate. The study had an additional exploratory objective, to compare the immune profiles and response seen in RA with innate immune profiles seen in healthy subjects prior to and subsequent to hepatitis B immunization. Although the RA and vaccine patients were not control matched, use of a common omics platform and bio-resource was initiated to enable a specific investigation of the innate immune response in RA, by comparison of the dysregulated immune response in early RA with a healthy innate immune response to the Hepatitis B antigen. To this end, we recruited a cohort of 267 early RA patients, and 52 healthy subjects receiving the hepatitis B vaccine. Following sample quality control exclusions we transcriptionally profiled 242 RA patients and 37 healthy vaccine recipients utilising whole blood, PBMCs, and CD14^+^, CD8^+^, and CD4^+^ leukocyte subsets at their first visit to the clinic and after 6 months, totalling 2257 unique samples. We ultimately followed these patients longitudinally for up to 18 months, collecting rich bio-samples and a range of clinical and omic data on the genome, transcriptome, proteome, metabolome and autoantibodies. The RA-MAP project has generated an unparalleled range of data and insights into the molecular heterogeneity of RA phenotypes in peripheral blood, which can serve as a fundamental reference for analysis of the blood immunological landscape in RA and other IMIDs.

## Methods

### Patient characteristics and study design

In the TACERA study, two hundred and seventy five patients were recruited of whom 270 fulfilled all eligibility criteria, that is newly diagnosed patients of at least 18 years of age with symptom duration less than 12 months, untreated with DMARDs or corticosteroids and who fulfilled either the 1987 American College of Rheumatology (ACR) or 2010 ACR/European League Against Rheumatism (EULAR) classification criteria for RA. Two eligible patients withdrew at baseline without providing any clinical information. A further patient who withdrew at baseline had some clinical information but insufficient to calculate disease activity scores. Therefore, for our cohort summary in Table 1 we describe baseline characteristics of these 267 remaining patients. Note that 239 of these 267 eligible patients had a 6-month assessment visit. All patients were seropositive at baseline: 93% were rheumatoid factor (RF) positive, and 87% were citrullinated protein antibody positive (ACPA). Subjects were recruited from 28 participating centres across the UK. Following enrolment subjects received treatment at the discretion of the supervising rheumatologist per the National Institute for Health and Clinical Excellence (NICE) guidelines for the management of RA in adults^13^. Patients were followed up for 18 months and seen every 3 months. Based on EULAR response criterion, 47.2% patients achieved a good response, 33.9% a moderate response and 18.9% showed no response. Clinical, laboratory, lifestyle, comorbidities and associated medication, patient reported outcome measures, and biological samples were collected at each visit. Blood samples for RNA extraction were taken at baseline and 6 months. Clinical data is summarised in Table 1. Ethical approval was authorised by the National Research Ethics Service London Central Committee (Reference number: 12/LO/0469). Informed, written consent was obtained from all study participants. Complete phenotypes and further details on the TACERA study are published elsewhere^14,15^.

**Table 1:** Baseline characteristics of patients in the TACERA study.

Characteristics	Baseline (n = 267)
Age, years	53.1 (15.2)
Female	192 (71.9%)
White Ethnicity	194 (72.7%)
BMI	
Female	27.53 (6.47)
Male	27.43 (4.97)
Overall	27.50 (6.08)
BMI Status	
Underweight: <18.5	9 (3.4%)
Healthy weight: (18.5,25)	85 (31.8%)
Overweight: (25,30)	95 (35.6%)
Obese: ≥30	78 (29.2%)
Smoking	
Never smoked	95 (35.6%)
Previous smoker	104 (39.0%)
Current smoker	68 (25.4%)
Alcohol consumption	
None	86 (32.3%)
1–5 units per week	115 (43.2%)
6–10 units per week	25 (9.4%)
11–15 units per week	11 (4.1%)
16–20 units per week	15 (5.7%)
More than 20 units per week	14 (5.3%)
Alcohol frequency	
Not Drinking	86 (32.2%)
1–2 days a year	28 (10.5%)
1–2 days a month	48 (18.0%)
1–2 days a week	58 (21.7%)
3–4 days a week	28 (10.5%)
5 days or more a week	19 (7.1%)
Rheumatoid Factor (RF) positive	247 (92.5%)
Anti-citrullinated protein antibody (ACPA) positive	230 (86.1%)
Disease Duration (years)	0.43 (0.23)
X-ray Larsen’s Score (hands and feet)	6.70 (8.76)
Charlson’s Comorbidity Index (original)	0.44 (0.84)
Charlson’s Comorbidity Index (2008)	0.81 (1.10)
SDAI	28.80 (14.29)
DAS28-CRP	4.85 (1.22)
Prescribed Medication	
Methotrexate (MTX)	202 (75.7%)
Hydroxychloroquine	141 (52.8%)
Leflunomide	0 (0.0%)
Sulfasalazine	18 (6.7%)
Oral glucocorticoids	17 (6.4%)
Parenteral glucocorticoids	126 (47.2%)
No RA medication	2 (0.7%)
Medication combinations prescribed	
No RA medication	2 (0.7%)
MTX only	51 (19.1%)
Other DMARDs only	20 (7.5%)
Oral glucocorticoids only	2 (0.7%)
Parenteral glucocorticoids only	15 (5.6%)
MTX & other DMARDs	53 (19.9%)
MTX & oral glucocorticoids	6 (2.2%)
MTX & parenteral glucocorticoids	33 (12.4%)
Other DMARDs & oral glucocorticoids	2 (0.7%)
Other DMARDs & parenteral glucocorticoids	23 (8.6%)
Oral & parenteral glucocorticoids	1 (0.4%)
MTX, other DMARDs & oral glucocorticoids	5 (1.9%)
MTX, other DMARDs & parenteral glucocorticoids	53 (19.9%)
MTX, oral & parenteral glucocorticoids	1 (0.4%)

Values are number (percentage) or mean (standard deviation). Abbreviations: BMI (Body Mass Index), SDAI (Simple disease activity index), DAS28-CRP (Disease Activity Score-28 for Rheumatoid Arthritis with C-Reactive Protein).

### Vaccine study design

Vaccine (Engerix B (recombinant Hepatitis B surface antigen)) recipients were recruited from healthcare workers receiving hepatitis B screening as part of their workplace induction at 4 participating centres across the UK (Newcastle, Birmingham, London and Glasgow). Subjects received Hepatitis B vaccination (20 micrograms Engerix B by IM injection at 0, 1 and 2 months), and were followed up for 8 visits (day -7, 0 (baseline), 1, 3, 7, 56, 57, 63). Clinical, lifestyle, demographics, and biological samples were collected at each visit. Blood samples for RNA extraction were taken at all 8 visits, with transcriptomics performed to the TACERA transcriptomic protocol on day -7, 0, 3, 56 and 63. Clinical data is summarised in Table 2. Informed, written consent was obtained from all study participants (study protocol is available on the RA-MAP figshare^16^).

**Table 2:** Baseline characteristics of Vaccine recipients.

Characteristics	Baseline (n = 52)
Age, years	32 (13.8)
Female	32 (61.5%)
White Ethnicity	45 (86.5%)
BMI	
Female	24.69 (5.91)
Male	25.84 (3.45)
Overall	25.08 (5.18)
BMI Status	
Underweight: <18.5	4 (7.7%)
Healthy weight: [18.5,25)	21 (40.4%)
Overweight: [25,30)	19 (36.5%)
Obese: ≥30	5 (9.6%)
No data	4 (7.7%)
Smoking	
Non-smoker	42 (80.8%)
Smoker	6 (11.5%)
No data	4 (7.7%)
Alcohol consumption	
No	6 (11.5%)
Yes	42 (80.8%)
No data	4 (7.7%)

Values are number (percentage) or mean (standard deviation).

### Patient biosampling, extraction, and biobanking

Biosamples, including blood, serum, plasma and urine were obtained from patients every 3 months for 18 months in the TACERA and Vaccine studies. The SOPs required that blood was drawn and stored locally for up to 30 minutes before transport to the local processing hub. According to SOPs serum, plasma and urine samples were processed no longer than 60 minutes after collection. PBMC processing, followed by isolation of CD4, CD8 and CD14 subsets, took place within 60 minutes of blood draw. Isolated cells were then lysed and frozen in QIAzol lysis reagent and stored at −80 °C for later RNA extraction. Samples were stored prior to RNA extraction until the last participant, last visit. For the whole blood RNA the blood was drawn into a Tempus tube and then incubated for 3 hours at room temperature then frozen at −20 °C for 24 hours before long term storage at −80 °C. Like the cell subsets RNA the whole blood RNA was extracted from these samples at the end of the study. Blood for RNA extraction was collected in Tempus blood RNA tubes (Applied Biosystems) and mixed by inverting. Additional blood sampling for isolation of PBMCs was collected into EDTA Vacutainer collection tubes (Becton Dickinson) and separated using Leucosep separation tubes (Greiner). Cell subsets were isolated from the peripheral blood using magnetic cell sorting. The Miltenyi MACS system was used to positively isolate CD14-expressing monocytes, CD4-expressing T cells and CD8-expressing T cells from the isolated PBMCs by following the manufacturer’s protocol (Miltenyi Biotech). The purified cells were lysed in QIAzol Lysis Reagent. Cell subset micro RNA was extracted using miRNeasy minikits (Qiagen) following the manufacturer’s protocol. Whole blood RNA was extracted from Tempus Blood tubes using MagMAX RNA isolation kits (Ambion) and then was subjected to removal of globin mRNA using GLOBINclear human 20 reaction kits (Ambion) following the manufacturer’s protocol. Multi-omic analysis of samples was performed at baseline and 6 months. All samples (n = 34,540) were deposited with UK Biocentre (Milton Keynes, UK) and are available to researchers on request to the RA-MAP sample access panel (https://research.ncl.ac.uk/ra-map/). Detailed SOPs are available in the RA-MAP figshare^16^. Some of the logistical processing challenges faced by the study are described elsewhere^12^.

### Microarray mRNA sample analysis

For microarray analysis, amplified RNA was hybridized to Illumina HumanHT-12 V4.0 expression beadchips and scanned on an Illumina Beadstation 500. Illumina’s GenomeStudio version 2011.1 with the Gene Expression Module v1.9.0 was used to generate signal intensity values. TACERA samples were randomized across the analysis plates, with samples from same RA subject (baseline and 6-month time points) assigned within same plate. Vaccine samples were run in separate batches, but also randomized across analysis plates. Non-normalised control and sample probe data was exported from GenomeStudio.

### mRNA transcriptome data analysis

To perform QC and exploratory data analysis (EDA), all mRNA sample data was imported to the R Limma package^17^ as a combined matrix to enable direct comparison of cell subsets. Where the goal is to isolate effects within a particular cell type, single cell type processing may be more optimal. Background correction and quantile normalization were performed using the Limma *neqc* function^18^, based on methods described by Shi *et al*.^19^. All probes with detection p-values of at most 0.05 in at least 100 samples were removed. This ensured that all remaining probes were expressed in a minimum of 100 samples, or just under 10% of all profiles. Probes were aggregated to gene level using the Limma *avereps* function to further reduce dimensionality and increase reproducibility^20^, using a simple mean aggregator as implemented in the limma package. After pre-processing, filters and exclusions, 18,562 genes were present in the analysis ready expression matrix. Dimensionality reduction by Principal component analysis (PCA) was used to check for outliers and unsupervised clustering effects. 12 samples were flagged as outliers in the dataset, we removed these from subsequent analysis (See full EDA and outlier markdown documents on GitHub).

### Small RNA sample and transcriptome data analysis

For Small RNA sequencing analysis, amplified small RNA was sequenced on an Illumina HtSeq. 2500 unit with single read flow cells to a depth of at least 10 million 50-bp reads per sample. TACERA samples were randomized across the analysis plates, with samples from same RA subject (baseline and 6-month time points) assigned within same plate. Adaptors were clipped off the reads using Trimmomatic^21^ (version 0.33) and then aligned to the GRCh38 genome using Bowtie-2 (version 2.3.0)^22^. Next, alignments to miRNA reference were counted with the htseq-count function from HTSeq (version 0.6.1p2)^23^ and the miRBase annotation release 22.1. Prior to normalization, transcripts in the resulting count table were filtered to a mean count per million (CPM) of at least 2, and normalised using the EdgeR CalcNormFactors function^24^.

### Plasma protein analyte analysis

Plasma samples were selected from 100 baseline patients with higher baseline disease activity (DAS28 > 4) who divided equally at the 6 month visit into 50 patients in remission (DAS28 < 2.6) and 50 with active disease (DAS28 > 4). Plasma samples from 40 healthy (vaccine) recipient (VC) subjects, were analyzed concurrently with the RA patient samples. 1310 analytes were measured in the selected plasma samples for baseline (RA, vaccine) and 6-month (RA) visits at SomaLogic, LLC (Boulder, CO USA) using SOMAscan v3.2 platform. RA and vaccine recipient samples were randomized across the analysis plates, with samples from same RA subject (baseline and 6-month time points) assigned within same plate. 124 analytes were flagged by the vendor for failing QC standards, leaving 1186 analytes available for analysis. Relative fluorescence unit (RFU) data were sequentially normalized for hybridization controls (internal standards per sample) to remove inter-run hybridization artifacts, median signal across all samples to remove other potential assay biases (assumes same total protein concentration across sample set), and calibration controls (common sample standards across analysis plates). The normalized RFU values were log_2_-transformed and then each analyte was independently 0-centered to the mean of the healthy subject cohort by shifting. 2 samples failed the vendor’s QC standards for median normalization scale factors within range of 0.4 to 2.5 and were excluded from further analysis (both 6-month samples from the active disease group).

### Auto-antibody sample analysis

501 serum samples were analysed from the TACERA cohort, comprising 265 baseline samples and 235 6-month follow-up samples. In parallel, 44 baseline and 38 follow-up samples from Vaccine recipients were measured. All samples were distributed on 96-well assay plates applying a randomised block design (timepoint, age, gender, healthy, RA).

A Luminex bead-based antigen array was produced (Protagen AG, Switzerland) to measure the autoantibody response against 192 human protein antigens. Antigens were selected based on literature data and autoantibody reactivity data of previous high-content profiling studies in RA and other rheumatic diseases. A subset of protein antigens (n = 46) were citrullinated *in vitro* using peptidyl arginine deiminase (PAD) to compare the autoantibody reactivity towards citrullinated and corresponding uncitrullinated antigens in early RA patients. Briefly, proteins were produced in *Escherichia coli* as His-tagged fusion proteins and purified by immobilised metal affinity chromatography. Coupling of antigens to magnetic carboxylated colour-coded beads (MagPlex microspheres, Luminex Corporation, Austin, Texas) was performed according to manufacturers’ protocols. Beads coupled with BSA, human IgG (hIgG), *E. coli* lysate and the eluate of vector only transformed *E. coli* were used as internal quality controls to evaluate the background reactivity, the measurement range or patient anti-*E. coli* reactivity, respectively. Finally, beads were combined and stored at 4–8 °C until use. An aliquot of the bead mix was incubated with the 1:100 diluted patient serum sample. Bound antibodies were measured following incubation with a secondary PE-labelled anti-human-IgG antibody in a FlexMap3D instrument (Luminex Corporation, Austin, Texas). The IgG reactivity values are given as median fluorescence intensity (MFI) and data of antigens fulfilling the minimum bead count criterion (>10 beads measured per bead ID) was used for data analysis. To monitor the inter-assay coefficient of variation, three in-process control samples were measured in triplicate on each 96-well assay plate using the autoantibody MFI values of all measured antigens. The overall median inter-plate CV was 7.7%. Evaluation of the control beads showed that the MFI values of control beads was as expected: The background reactivity towards BSA was 4 MFI, the reactivity to the *E. coli* lysate was 8 MFI, to the vector only eluate 6 MFI and to hIgG 22,000 MFI. The hIgG coupled bead was used to confirm the reactivity of the PE-conjugated detection antibody. To obtain reliable MFI values for data analysis the bead count statistics of the autoantibody data were evaluated. The median bead count of all samples was 167, with 0.01% of all samples having a bead count <10 and 0.9% of all samples having a bead count of <35. All samples and antigens met the bead-count criterion.

### Auto-antibody data analysis

Raw Luminex autoantibody measurement values were processed and analysed using the R programming language (http://www.r-project.org/ version 3.3.0) and KNIME 3.2 (https://www.knime.org/) to produce text CSV files for quality control and further statistical analysis. The main pre-processing steps were removal of data points that did not pass the quality control with regard to bead count criterion, MFI values were transformed into log2 values. Following the exclusion of autoantibodies with <10% seropositivity in RA patients, 163 autoantibodies were retained for analysis. To adjust for systematic variation in the overall MFI values of individual samples, the data were median-centred by the sample.

### Metabolomic sample preparation

After thawing, TACERA serum samples were centrifuged at high speed (13000 ×x g_(av)_) and then filtered by centrifuging at 10,000 × g_(av)_ through a thoroughly pre-washed 3000 molecular weight cut-off filter (Pall, Omega 3k) to remove proteins which greatly improves the quality of the subsequent NMR spectra^25^. The filtrate was diluted 1:4 with D2O/H2O (40%) containing NaCl (150 mM), deuterated 4,4-dimethyl–4-silapentane-1-sulfonic acid (D6-DSS) (2 mM) as a chemical shift standard, difluorotrimethylsilanyl phosphonic acid (DFTMP) (0.4 mM) as a pH indicator^26^ as suggested by Chenomx Inc, sodium azide (0.4%) and sodium phosphate (100 mM) pH 7.0. The samples were then stored frozen at −80 °C until analysed, at which time they were thawed and a sample (35 µl) transferred to a 1.7 mm NMR tube which was then capped. Urine samples were thawed, centrifuged and diluted 1:4 with the NMR buffer detailed above. Samples were then carefully pH adjusted to 7.0 until stable^27^ and then frozen at −80 °C until analysed.

### Urine and Serum metabolomics data analysis

One-dimensional 1H NMR spectra were acquired at 300 K using a NOESY pulse sequence including water suppression with pre-saturation on a Bruker DRX 600 MHz NMR spectrometer equipped with a TXI 1.7 mm cryoprobe. 2D homonuclear (1J-resolved) spectra were also acquired for each sample and heteronuclear (1H_1H_13H TOCSY and C HSQC) spectra were also recorded for selected samples to aid spectral assignment. Samples were processed and data calibrated with respect to the DSS signal. Spectra were read into Metabolab^28^, in Matlab (version 2016a, The Mathworks, Natick, MA), and spectra were segmented into 0.006-ppm (3 Hz) chemical shift ‘bins’ and the spectral area within each bin was integrated. Spectra were corrected for baseline offset using a spline fit and then normalised using Probabilistic Quotient Normalisation (PQN)^29,30^ and a generalised log transformation was applied^31,32^. Binned data were then compiled into a matrix, with each row representing an individual sample. NMR databases (Human Metabolome Database version 3) and the Chenomx NMR suite (Chenomx, Professional version 8.0)^33^ were used to identify and quantify metabolites present in each sample. Automated metabolite identification with Chenomx was used to produce an initial fit and then manual fitting of the Chenomx provided metabolite spectral library was done. This focussed on a set of metabolites previously identified as being present in human serum^34^ and urine^35^. Other published data on metabolites identified in human sera were also used to guide identification^36,37^. Following automated and manual metabolite identification, 40 known metabolites were identified and quantified in sera and 42 in urine.

### Genotyping

Genotyping was performed using the Illumina HumanCoreExome-24-v1-0 (Batch 1) or Illumina InfiniumCoreExome-24-v1-1 (Batch 2) according to the manufacturer’s SOP. Raw intensity data files (idat format) from the Illumina iScan instrument were imported into GenomeStudio (v2011.1). Samples <90% call rate were excluded. Data was exported to PLINK PED/MAP format on the forward strand. Data was converted from PED/MAP to BED/BIM/FAM using PLINK v1.07. HumanCoreExome-24v1-0_A_PopulationReport_MAF_022015.txt or InfiniumCoreExome-24v1-1_A_PopulationReport_MAF.txt was used to obtain a list of all variants on the array with a MAF >0.005. This list was used to extract the variants from the genotype file. Variants with a GenomeStudio Cluster Separation <0.3 were excluded. Variants with <98% call rate were excluded. Individuals with <98% call rate were excluded. Palindromic SNPs (AT/CG) were excluded and the file split per chromosome. Variant IDs were updated to match Haplotype Reference Consortium v1.1 using GenotypeHarmonizer. SNPs in common between the two arrays were extracted from each dataset and the files combined (TACERA_combined). W.Rayner’s script HRC-1000G-check-bim-4.23.pl was used to align SNPs to HRC v1.1 panel. PLINK v1.9 was used to convert the PLINK files to VCF. The Wellcome Trust Sanger Institute imputation server was used to impute the data to the HRC v1.1 panel using SHAPEIT2 for phasing and PBWT for imputation.

### HLA imputation methods

HLA imputation was performed using PLINK to extract 28Mb-34Mb on chromosome 6. SNP2HLA was used for imputation with the Type 1 Diabetes Genetics Consortium (T1DGC) Panel^38^. Custom bash and STATA scripts were used to extract the HLA haplotypes from the bgl.phased file.

## Data Records

**Fig. 2:**
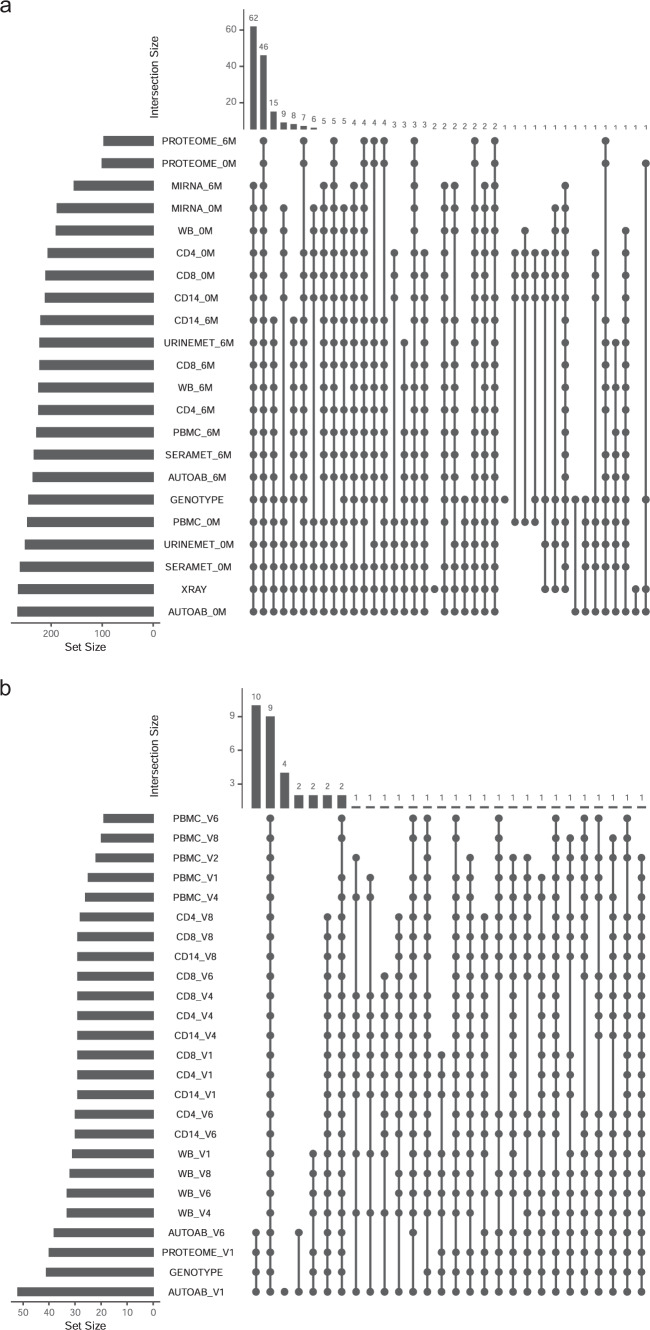
Summary of sample-platform overlap in (**a**) The TACERA cohort, (**b**) The Vaccine Cohort. Vaccine V1-V8 relates to visits 1, 2, 4, 6 & 8 at day -7, 0, 3, 56, 63.

Figure 2 summarises the number of samples processed on each omic platform. All raw data and processed mRNA and small RNA data are available in the NCBI GEO series accession number GSE97476^39^. Serum and urine metabolomic data have been deposited at the MetaboLights database of the European Bioinformatics Institute (EBI) under MTBLS1497^40^. Genotype data has been deposited in the European Genome-phenome Archive (EGA) with ID number EGAS00001004424^41^. De-Identified clinical data, X-ray, Somalogic proteomic data, including aptamer annotation and Protagen autoantibody data are available in the RA-MAP project in figshare^16^.

## Technical Validation

### Exploratory data analysis across omic platforms

**Fig. 3:**
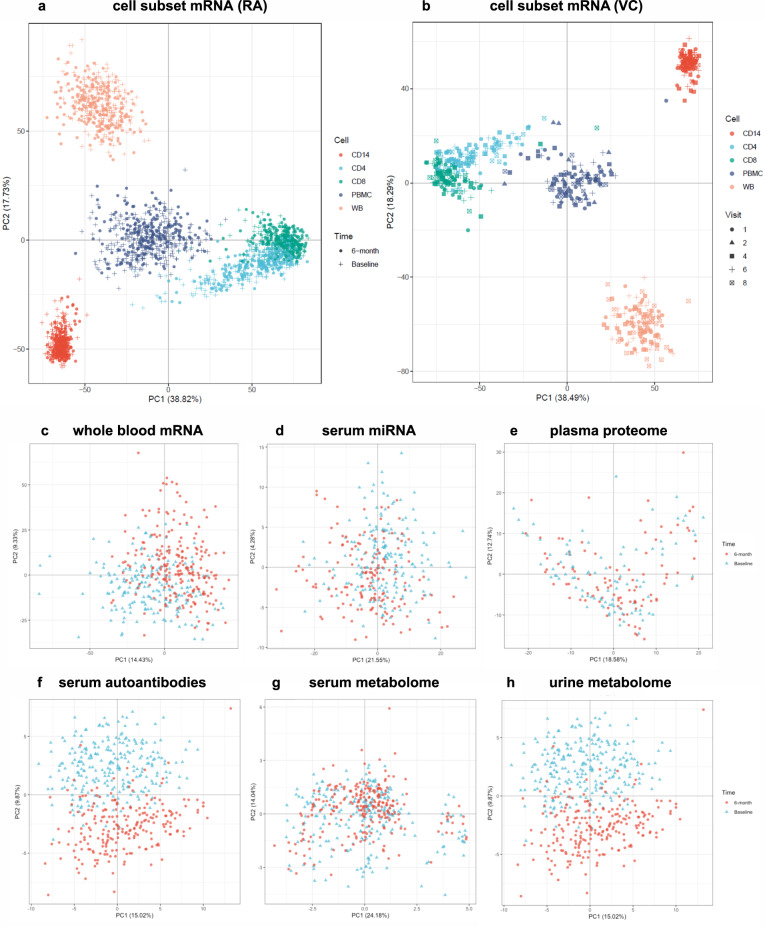
Principal component analysis (PCA) across multiple omic platforms at baseline and 6-months in whole blood and cell subsets in (**a**) the TACERA early RA cohort and (**b**) across 6 visits (day −7, 0, 3, 56 and 63) in the vaccine cohort. Similar separation by cell type is evident in both RA and vaccine cohorts. Some evidence of separation by time is seen in the RA cohort, but this is less evident in the Vaccine cohort. Multi-omic PCA comparison at baseline and 6-months in early RA across (**c**) whole blood mRNA, (**d**) micro RNA (serum), (**e**) proteome (plasma), (**f**) autoantibodies (serum), (**g**) metabolome (serum) and (**h**) metabolome (urine). Clear separation with time is seen in Whole Blood and cell subset mRNA, Serum miRNA, Serum Autoantibodies and Urine metabolome.

**Fig. 4:**
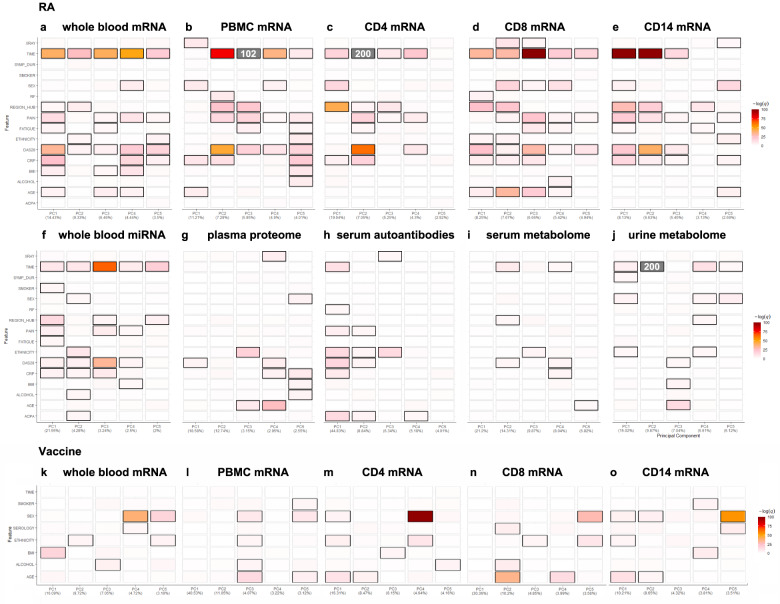
Unsupervised PCA Driver analysis of multi-omic compartments across TACERA early RA patients and Hepatitis B vaccine recipients, showing clinical features and their degree of association with Principal Components (PC) 1–5, percentage indicating variation accounted for by the PC and with coloring indicating the –log q-value of the association (scaled to maximum range of PC1 across all compartments, off scale associations are indicated in grey with –log q written). Significant drivers with an FDR threshold of 5% are indicated by outline. Specific drivers of variation in expression in analysed samples are indicated in TACERA patients in (**a**) Whole Blood mRNA, (**b**) PBMC mRNA, (**c**) CD4 mRNA, (**d**) CD8 mRNA, (**e**) CD14 mRNA, (**f**) Whole Blood micro RNA, (**g**) Plasma proteomics, (**h**) Serum autoantibodies, (**i**) Serum metabolome, (**j**) Urine metabolome. In VACCINE recipients in (**k**) Whole Blood mRNA, (**l**) PBMC mRNA, (**m**) CD4 mRNA, (**n**) CD8 mRNA, (**o**) CD14 mRNA. Clinical features include XRAY (quantitative measure of bone erosion at sample timepoint (0 or 6 m)), TIME (sample annotation at baseline or 6-months), SYMP_DUR (Symptom duration at diagnosis), SMOKER (smoking status Y, N, Previous), SEX (M/F), RF (Rheumatoid factor positive Y/N), PAIN (quantitative measure of pain at sample timepoint (0 or 6 m)), FATIGUE (quantitative measure of fatigue at sample timepoint (0 or 6 m)), ETHNICITY (Ethnic origin), DAS28 (disease activity score in 28 joints at sample timepoint (0 or 6 m)), CRP (quantitative measure of C-reactive protein at sample timepoint (0 or 6 m)), BMI (Body Mass Index at baseline), ALCOHOL (Y/N), AGE (Age at baseline), ACPA (anti-citrullinated protein antibody positive Y/N), SEROLOGY (Hepatitis B serology at week 9). In the TACERA cohort in 3a-j the first two PCs are closely associated with DAS28 scores in Whole Blood, PBMC mRNA, CD4 mRNA, and CD14 mRNA; and rather less closely associated with the other platforms. In 3k-o, in contrast to the highly dysregulated immune system seen in RA patients, the biological perturbation following a vaccine-related immune challenge in the healthy volunteers appears negligible.

Whole blood, cell subsets and serum readouts from each individual omic data platform were compared at baseline and six months, using a unified exploratory data analysis (EDA) approach employing a range of multi-dimensional visualisation and dimensionality reduction methods implemented in the bioplotr package (https://github.com/dswatson/bioplotr). The technical validity of each platform was explored using visualisations of mean variance / dispersion plots, density plots, subject similarity and principal component analysis (PCA). Full EDA markdown documents and data files for each platform are available in the RA-MAP GitHub. In Fig. 3, PCA is used to gain an overview of each omics platform at baseline and 6-months in whole blood and cell subsets in the TACERA early RA cohort (a, c-h) and across a range of time points in the vaccine cohort (b, k-o). Similar separation by cell type is evident in both RA and vaccine cohorts. After QC, including limited outlier removal, all Omics platforms appear relatively homogeneous with no unexpected structure in PCA projections. Some evidence of separation by time is seen in the RA cohort in whole blood and cell subset mRNA, serum miRNA, serum autoantibodies and urine metabolomics. Clear time separation is less evident in the Vaccine cohort. Collectively the EDA across each platform provides consistent evidence of technical validation. In order to identify those clinical features driving the observed multi-omic changes, giving some biological validation to the data, we performed unsupervised PCA Driver analysis (Fig. 4) which shows the degree of association between Principal Components (PC1-5; % PC effect indicated) and clinical variables in each multi-omic platform. The driver plot heatmap indicates the –log q-value of the association with each clinical and technical variable. Significant drivers are indicated by outline with an FDR threshold of 5%. Unlike the relatively non-specific PCA projections, PC driver plots allow direct evaluation of influence of different clinical and technical variables on variation of expression in the samples. In the TACERA cohort, the larger PCs, representing the largest source of variance, are closely associated with time and DAS28 and related disease activity scores in whole blood and cell subset mRNA and whole blood miRNA; and rather less closely associated with the other platforms. Notably time and DAS28 correlate closely with the exception of the urine metabolome where changes in time, including the joint strongest association seen in the dataset, are less well correlated with disease activity. This leads us to conclude that changes over time in the urine metabolome driven by PC2 are unlikely to be disease related. We note that region hub shows some correlation with measures of disease activity in some Omic platforms. Samples from 28 patient recruiting centres were processed in 7 regional hubs^14^, we hypothesize that this may reflect a higher proportion of more severe RA cases seen in tertiary referral centres. In the Vaccine cohort, in contrast to the highly dysregulated immune system seen in RA patients, the disturbance following a vaccine related immune challenge in the healthy volunteers appears negligible.

Collectively insights from EDA in the TACERA and Vaccine cohorts support the high technical quality of both data sets. Principal Component Driver analysis provides biological evidence of disease signatures in the TACERA cohort that are guiding our ongoing analysis of this dataset.

## Usage Notes

Genotype data is made available through the European Genome-phenome Archive (EGA). Request for data access will be referred directly to our Data Access Committee: https://ega-archive.org/datasets/EGAD00001006736. If you need to request access to this data set, please contact the RA-MAP Data Access Committee (Contact person:m.r.barnes@qmul.ac.uk). Applicants will be asked to complete the Data Access Agreement (DAA) (including a brief summary of the proposal, proposed usage of the dataset, the storage of data, so the DAC can determine if the planned usage falls within the consents) and to agree to the terms and conditions of the DAA. The DAA must be signed by the applicant and the relevant Head of Department, or equivalent. If applications include a named collaborator then the collaborator’s Institution must sign and submit a separate DAA. A template DAA can be found on the EGA website: https://ega-archive.org/submission/dac/documentation.

## Supplementary information

Supplementary Table 1

## Data Availability

Fully annotated Executable R scripts and R Markdown documents are available in our public RA-MAP GitHub in order to allow complete reproduction of our analysis workflow (https://github.com/C4TB/RA-MAP). All analyses were conducted in R version 4.0.5.
