# Supplementary material for: RA-MAP, molecular immunological landscapes in early rheumatoid arthritis and healthy vaccine recipients
Source: Sci Data. 2022 May 9;9:196. doi: 10.1038/s41597-022-01264-y (PMC9085807; doi:10.1038/s41597-022-01264-y)
Supplement: Supplementary file 1 — Supplementary Table 1 [file 41597_2022_1264_MOESM1_ESM.docx]

Supplementary Table 1. RA-MAP Consortium Members and Competing Interests

| **Name** | **Affiliation** | **Please provide details of contribution** | **Competing interest statement (if any)** |
| --- | --- | --- | --- |
| Adwoa Hughes-Morley | Centre for Musculoskeletal Research, School of Biological Sciences, Faculty of Biology, Medicine and Health, University of Manchester NIHR Manchester Musculoskeletal Biomedical Research Unit, Central Manchester NHS Foundation Trust, Manchester Academic Health Science Centre | Contributed to the acquisition, analysis and interpretation of data; critical review of the manuscript | None |
| Alexandra Walker | R&D GlaxoSmithKline, Stevenage, United Kingdom | Contributed to concept and design of study; interpretation of the data; critical review of the manuscript | Employee of GSK |
| Alexandru Cuza | UCB Celltech, Slough, United Kingdom | Contributed to concept and design of study; interpretation of the data; critical review of the manuscript | Employee of UCB |
| Amaya Gallagher-Syed | Centre for Translational Bioinformatics, William Harvey Research Institute, Faculty of Medicine and Dentistry, Queen Mary University of London , London , UK | Contributed to concept and design of study; interpretation of the data; critical review of the manuscript | None |
| Amy Anderson | NIHR Newcastle Biomedical Research Centre in Ageing and Long-term Conditions, Institute of Cellular Medicine, Newcastle University and Musculoskeletal Unit, Newcastle upon Tyne Hospitals NHS Foundation Trust | Contributed to concept and design of study; interpretation of the data; critical review of the manuscript | None |
| Andrea Haynes | R&D GlaxoSmithKline, Stevenage, United Kingdom | Contributed to concept and design of study; interpretation of the data; critical review of the manuscript | Employee of GSK |
| Andrew Filer | University of Birmingham and University Hospitals Birmingham NHS Trust and Sandwell and West Birmingham Hospitals NHS Trust | Contributed to concept and design of study; interpretation of the data; critical review of the manuscript | None |
| Andrew Long | Abbvie Bioresearch Center Inc., Worcester, MA, USA | Contributed to concept and design of study; interpretation of the data; critical review of the manuscript | Employee of Abbvie Inc |
| Andrew P. Cope | Academic Department of Rheumatology, Division of Immunology, Infection and Inflammatory Disease, Faculty of Life Sciences, King’s College London | Workpackage Lead for this study: contributions to study set up, acquisition and provision of datasets and analysis, and critical review of the manuscript | None |
| Angela Parke | Academic Department of Rheumatology, Division of Immunology, Infection and Inflammatory Disease, Faculty of Life Sciences, King’s College London | Contributed to concept and design of study; interpretation of the data; critical review of the manuscript | None |
| Anthony Rowe | Janssen Research & Development | Contributed to concept and design of study; interpretation of the data; critical review of the manuscript | Employee of Janssen |
| Arnaud Didierlaurent | R&D GlaxoSmithKline, Stevenage, United Kingdom | Contributed to concept and design of study; interpretation of the data; critical review of the manuscript | Employee of GSK |
| Ashley Gilmour | Institute of Infection, Immunity and Inflammation, College of Medical, Veterinary and Life Sciences, University of Glasgow, Glasgow, UK | Contributed to concept and design of study; interpretation of the data; critical review of the manuscript | None |
| Athula Herath | Respiratory, Inflammation & Autoimmunity, MedImmune Ltd, Cambridge, UK | Contributed to concept and design of study; interpretation of the data; critical review of the manuscript | Employee of Medimmune Ltd |
| Ayako Wakatsuki Pedersen | Institute of Cellular Medicine, Newcastle University | RA-MAP project manager: Overall management and facilitate the process of data acquisition and analysis | None |
| Aysin Tulunay Virlan | Institute of Infection, Immunity and Inflammation, College of Medical, Veterinary and Life Sciences, University of Glasgow, Glasgow, UK | Contributed to concept and design of study; interpretation of the data; critical review of the manuscript | None |
| Ben Allen | Bioinformatics Support Unit, Faculty of Medical Sciences, Newcastle University, Newcastle Upon Tyne, UK | Contributed to concept and design of study; interpretation of the data; critical review of the manuscript | None |
| Benjamin A Fisher | Institute of Inflammation and Ageing, University of Birmingham and NIHR Biomedical Research Centre, University Hospitals Birmingham NHS Trust | Contributed to analysis and interpretation of data, and drafting and critical review of the manuscript. | None |
| Blerina Kola | Medical Director Inflammation, Pfizer Ltd, Walton Oaks, Walton on the Hill, Tadworth, Surrey, KT20 7NS, U.K | Collection and integration of patient level data; critical review of manuscript | Pfizer employee and Pfizer shareholder |
| Bohdan Harvey | Abbvie Bioresearch Center Inc., Worcester, MA, USA | Contributed to concept and design of study; interpretation of the data; critical review of the manuscript | Employee of Abbvie Inc |
| Brian Tom | MRC Biostatistics Unit, University of Cambridge, UK | Design of the work, data acquisition and cleaning, statistical analysis, interpretation and writing; approval of final version of manuscript and accountable for all aspects of the work | None |
| Carl S. Goodyear | Institute of Infection, Immunity and Inflammation, College of Medical, Veterinary and Life Sciences, University of Glasgow, Glasgow, UK | Contributed to concept and design of study; interpretation of the data; critical review of the manuscript | None |
| Carolyn Cuff | Abbvie Bioresearch Center Inc., Worcester, MA, USA | Contributed to concept and design of study; interpretation of the data; critical review of the manuscript | Employee of Abbvie Inc |
| Catharien Hilkens | NIHR Newcastle Biomedical Research Centre in Ageing and Long-term Conditions, Institute of Cellular Medicine, Newcastle University and Musculoskeletal Unit, Newcastle upon Tyne Hospitals NHS Foundation Trust | Contributed to concept and design of study; interpretation of the data; critical review of the manuscript | None |
| Catharina Lindholm | BioPharmaceuticals R&D, Astrazeneca, Sweden | Contributed to concept and design of study; interpretation of the data; critical review of the manuscript | Employee of Astrazeneca |
| Catherine T Mela | Roche Products Ltd. 6 Falcon Way, Shire Park, Welwyn Garden City, AL7 1TW. UK | Contributed to concept and design of study; interpretation of the data; critical review of the manuscript | Employee of Roche Products ltd at the time of the study |
| Christopher D Buckley | The Kennedy Institute of Rheumatology, University of Oxford, Oxford, UK | Contributed to concept and design of study; interpretation of the data; critical review of the manuscript | CDB is a founder of Mestag and holds founder shares |
| Chris Larminie | R&D GlaxoSmithKline, Stevenage, United Kingdom | Contributed to concept and design of study; interpretation of the data; critical review of the manuscript | Employee of GSK |
| Chris Marshall | Global Medicines Development, Astrazeneca | Contributed to concept and design of study; interpretation of the data; critical review of the manuscript | Employee of Astrazeneca |
| Christopher John | Centre for Experimental Medicine and Rheumatology, William Harvey Research Institute, Faculty of Medicine and Dentistry, Queen Mary University of London, London, UK | Contributed to concept and design of study; interpretation of the data; critical review of the manuscript | None |
| Christopher M Mela | Roche Products Ltd. 6 Falcon Way, Shire Park, Welwyn Garden City, AL7 1TW. UK. | Contribution to acquisition, analysis and interpretation of data; critical review of manuscript | CM Mela was an employee of Roche Products Ltd. at the time of the study |
| Claudio Carini | Inflammation and Immunology RU, Worldwide Research & Development Pfizer Inc. | Provision of strategic input and patient level data; critical review of manuscript | None |
| Costantino Pitzalis | Centre for Experimental Medicine and Rheumatology, William Harvey Research Institute, Faculty of Medicine and Dentistry, Queen Mary University of London, London, UK | Contributed to concept and design of study; interpretation of the data; critical review of the manuscript | None |
| Coziana Ciurtin | Centre for Adolescent Rheumatology, Division of Medicine, University College London, London, UK | Contributed to concept and design of study; interpretation of the data; critical review of the manuscript | None |
| Dan Baker | Janssen Research & Development | Contributed to concept and design of study; interpretation of the data; critical review of the manuscript | Employee of Janssen |
| Daniel Ziemek | Inflammation and Immunology RU, Worldwide Research & Development Pfizer Inc. | Contributed to concept and design of study; interpretation of the data; critical review of the manuscript | Employee of Pfizer |
| Daniela Dastros-Pitei | Eisai Ltd, European Knowledge Centre, Mosquito Way, Hatfield, Herts, AL10 9SN | Contributed to concept and design of study; interpretation of the data; critical review of the manuscript | Employee of Eisai at the time of the study |
| Dao Nguyen | University College London (Division of Medicine) | Contributed to concept and design of study; interpretation of the data; critical review of the manuscript | None |
| David L. Scott | Academic Department of Rheumatology, Division of Immunology, Infection and Inflammatory Disease, Faculty of Life Sciences, King’s College London | Contributed to the data acquisition and critical review of the manuscript | None |
| David S Watson | Centre for Translational Bioinformatics, William Harvey Research Institute, Faculty of Medicine and Dentistry, Queen Mary University of London , London , UK | Contributed to concept and design of study; interpretation of the data; critical review of the manuscript | None |
| Deborah Symmons | Centre for Musculoskeletal Research, School of Biological Sciences, Faculty of Biology, Medicine and Health, University of Manchester NIHR Manchester Musculoskeletal Biomedical Research Unit, Central Manchester NHS Foundation Trust, Manchester Academic Health Science Centre | Contributed to the conception and design of the work; drafted and critical revision of the manuscript | None |
| Dennis Lendrem | NIHR Newcastle Biomedical Research Centre in Ageing and Long-term Conditions, Translational & Clinical Research Institute, Newcastle University and Musculoskeletal Unit, Newcastle upon Tyne Hospitals NHS Foundation Trust | Contributed to concept and design of study; interpretation of the data; critical review of the manuscript | None |
| Denny Verbeeck | Janssen Research & Development | Contributed to concept and design of study; interpretation of the data; critical review of the manuscript | Employee of Janssen |
| Desmond Padhji | Department of Neuroscience, Amgen, Inc, Thousand Oaks, CA 91320, USA | Contributed to concept and design of study; interpretation of the data; critical review of the manuscript | Employee of Amgen Inc |
| Donna Finch | Respiratory, Inflammation & Autoimmunity, MedImmune Ltd, Cambridge, UK | Contributed to concept and design of study; interpretation of the data; critical review of the manuscript | Employee of Medimmune Ltd |
| Duncan Porter | Gartnavel General Hospital, Glasgow, UK | Contributed to concept and design of study; interpretation of the data; critical review of the manuscript | None |
| Emma Vernon | Roche Products Ltd. 6 Falcon Way, Shire Park, Welwyn Garden City, AL7 1TW. UK | Contributed to concept and design of study; interpretation of the data; critical review of the manuscript | Employee of Roche Products ltd at the time of the study |
| Faye Cooles | NIHR Newcastle Biomedical Research Centre in Ageing and Long-term Conditions, Institute of Cellular Medicine, Newcastle University and Musculoskeletal Unit, Newcastle upon Tyne Hospitals NHS Foundation Trust | Contributed to concept and design of study; interpretation of the data; critical review of the manuscript | None |
| Feng Hong | Abbvie Bioresearch Center Inc., Worcester, MA, USA | Contributed to concept and design of study; interpretation of the data; critical review of the manuscript | Employee of Abbvie Inc |
| Fiona Clarke | Academic Department of Rheumatology, Division of Immunology, Infection and Inflammatory Disease, Faculty of Life Sciences, King’s College London | Contributed to concept and design of study; interpretation of the data; critical review of the manuscript | None |
| Fiona Stirling | University of Manchester (Centre for Musculoskeletal Research, School of Biological Sciences, Faculty of Biology, Medicine and Health, University of Manchester | Contributed to concept and design of study; interpretation of the data; critical review of the manuscript | None |
| Fowzia Ibrahim | Academic Department of Rheumatology, Division of Immunology, Infection and Inflammatory Disease, Faculty of Life Sciences, King’s College London | Contributed to concept and design of study; interpretation of the data; critical review of the manuscript | None |
| Frances Humby | Centre for Experimental Medicine and Rheumatology, William Harvey Research Institute, Faculty of Medicine and Dentistry, Queen Mary University of London, London, UK | Contributed to concept and design of study; interpretation of the data; critical review of the manuscript | None |
| Francisco Bonachela Capdevila | Janssen Research & Development | Contributed to concept and design of study; interpretation of the data; critical review of the manuscript | Employee of Janssen |
| Frederic Geissmann | Department of Immunobiology, Division of Immunology, Infection and Inflammatory Disease, Faculty of Life Sciences, King’s College London | Contributed to concept and design of study; interpretation of the data; critical review of the manuscript | None |
| Frederique Ponchel | Leeds Institute of Rheumatic and Musculoskeletal Medicine, | Contribution to the study concept; critical revision | None |
| Gemma Molyneux | Academic Department of Rheumatology, Division of Immunology, Infection and Inflammatory Disease, Faculty of Life Sciences, King’s College London | Contributed to concept and design of study; interpretation of the data; critical review of the manuscript | None |
| Gemma Simpson | Academic Department of Rheumatology, Division of Immunology, Infection and Inflammatory Disease, Faculty of Life Sciences, King’s College London | Contributed to concept and design of study; interpretation of the data; critical review of the manuscript | None |
| Georgina Thorborn | Centre for Experimental Medicine and Rheumatology, William Harvey Research Institute, Faculty of Medicine and Dentistry, Queen Mary University of London, London, UK | Contributed to concept and design of study; interpretation of the data; critical review of the manuscript | None |
| Gerry Parker | UCB Pharma | Provision of strategic input and patient level data; critical review of manuscript | None |
| Gioia Altobelli | University College London (Division of Medicine) | Contributed to concept and design of study; interpretation of the data; critical review of the manuscript | None |
| Graham R Smith | Bioinformatics Support Unit, Faculty of Medical Sciences, Newcastle University, Newcastle Upon Tyne, UK | Contributed to concept and design of study; interpretation of the data; critical review of the manuscript | None |
| Hannah Edwards | UCB Celltech, Slough, United Kingdom | Contributed to concept and design of study; interpretation of the data; critical review of the manuscript | Employee of UCB |
| Hannah Tipney | R&D GlaxoSmithKline, Stevenage, United Kingdom | Contributed to concept and design of study; interpretation of the data; critical review of the manuscript | Employee of GSK |
| Hans-Dieter Zucht | Protagen AG, 44227 Dortmund, Otto-Hahn Street 15, Germany | Contributed to concept and design of study; interpretation of the data; critical review of the manuscript | Employee of Protagen AG |
| Hayley Noble | Academic Department of Rheumatology, Division of Immunology, Infection and Inflammatory Disease, Faculty of Life Sciences, King’s College London | Contributed to concept and design of study; interpretation of the data; critical review of the manuscript | None |
| Heidi Lempp | Academic Department of Rheumatology, Division of Immunology, Infection and Inflammatory Disease, Faculty of Life Sciences, King’s College London | Contributed to concept and design of study; interpretation of the data; critical review of the manuscript | None |
| Humayara Ali | University College London (Division of Medicine) | Contributed to concept and design of study; interpretation of the data; critical review of the manuscript | None |
| Iain B. McInnes | Institute of Infection, Immunity and Inflammation, College of Medical, Veterinary and Life Sciences, University of Glasgow, Glasgow, UK | Contribution to the study concept; critical revision of manuscript | None |
| Ian C. Scott | Academic Department of Rheumatology, Division of Immunology, Infection and Inflammatory Disease, Faculty of Life Sciences, King’s College London | Contributed to the data acquisition and critical review of the manuscript | None |
| Ian N Bruce | University of Manchester (Centre for Musculoskeletal Research, School of Biological Sciences, Faculty of Biology, Medicine and Health, University of Manchester | Contributed to concept and design of study; interpretation of the data; critical review of the manuscript | None |
| Iona Donnelly | Institute of Infection, Immunity and Inflammation, College of Medical, Veterinary and Life Sciences, University of Glasgow, Glasgow, UK | Contributed to concept and design of study; interpretation of the data; critical review of the manuscript | None |
| Ivana Vranic | Roche Products Ltd. 6 Falcon Way, Shire Park, Welwyn Garden City, AL7 1TW. UK | Contributed to concept and design of study; interpretation of the data; critical review of the manuscript | Employee of Roche Products ltd at the time of the study |
| James A. Butler | SimOmics Ltd, Kennedy Institute of Rheumatology, University of Oxford, Roosevelt Drive, Headington, Oxford, OX3 7FY, UK | Contributed to concept and design of study; interpretation of the data; critical review of the manuscript | Employee of Simomics |
| James Galloway | Academic Department of Rheumatology, Division of Immunology, Infection and Inflammatory Disease, Faculty of Life Sciences, King’s College London | Contributed to concept and design of study; interpretation of the data; critical review of the manuscript | None |
| Jamie C Sergeant | University of Manchester (Centre for Musculoskeletal Research, School of Biological Sciences, Faculty of Biology, Medicine and Health, University of Manchester | Contributed to concept and design of study; interpretation of the data; critical review of the manuscript | None |
| Jane Worthington | University of Manchester (Centre for Musculoskeletal Research, School of Biological Sciences, Faculty of Biology, Medicine and Health, University of Manchester | Contributed to concept and design of study; interpretation of the data; critical review of the manuscript | None |
| Jehan El-Jawhari | Leeds Institute of Rheumatic and Musculoskeletal Medicine, University of Leeds | Contributed to concept and design of study; interpretation of the data; critical review of the manuscript | None |
| Jessica Tarn | NIHR Newcastle Biomedical Research Centre in Ageing and Long-term Conditions, Institute of Cellular Medicine, Newcastle University and Musculoskeletal Unit, Newcastle upon Tyne Hospitals NHS Foundation Trust | Contributed to concept and design of study; interpretation of the data; critical review of the manuscript | None |
| Joanne Ellis | R&D GlaxoSmithKline, Stevenage, United Kingdom | Contributed to concept and design of study; interpretation of the data; critical review of the manuscript | Employee of GSK |
| John Casement | Bioinformatics Support Unit, Faculty of Medical Sciences, Newcastle University, Newcastle Upon Tyne, UK | Contributed to concept and design of study; interpretation of the data; critical review of the manuscript | None |
| John Isaacs | NIHR Newcastle Biomedical Research Centre in Ageing and Long-term Conditions, Institute of Cellular Medicine, Newcastle University and Musculoskeletal Unit, Newcastle upon Tyne Hospitals NHS Foundation Trust | RA-MAP Consortium Academic Lead: contribution to the design of the study; critical review and approval of manuscript | None |
| Julie Diboll | NIHR Newcastle Biomedical Research Centre in Ageing and Long-term Conditions, Institute of Cellular Medicine, Newcastle University and Musculoskeletal Unit, Newcastle upon Tyne Hospitals NHS Foundation Trust | Contributed to concept and design of study; interpretation of the data; critical review of the manuscript | None |
| Karim Raza | University of Birmingham and University Hospitals Birmingham NHS Trust and Sandwell and West Birmingham Hospitals NHS Trust | Contributed to concept and design of study; interpretation of the data; critical review of the manuscript | None |
| Katriona Goldmann | Centre for Experimental Medicine and Rheumatology, William Harvey Research Institute, Faculty of Medicine and Dentistry, Queen Mary University of London, London, UK | Contributed to concept and design of study; interpretation of the data; critical review of the manuscript | None |
| Kirsty Hicks | Statistics and Programming, Pharma R&D, GlaxoSmithKline | Contributed to study design; statistical analyses of data; critical review of the manuscript | Shareholder in GSK |
| Liliane Fossati-Jimack | Centre for Experimental Medicine and Rheumatology, William Harvey Research Institute, Faculty of Medicine and Dentistry, Queen Mary University of London, London, UK | Contributed to concept and design of study; interpretation of the data; critical review of the manuscript | None |
| Lucy Rowell | Roche Products Ltd. 6 Falcon Way, Shire Park, Welwyn Garden City, AL7 1TW. UK | Contributed to concept and design of study; interpretation of the data; critical review of the manuscript | Employee of Roche Products ltd |
| Marc Levesque | Abbvie Bioresearch Center Inc., Worcester, MA, USA | Contributed to concept and design of study; interpretation of the data; critical review of the manuscript | Employee of Abbvie Inc |
| Mark C. Coles | SimOmics Ltd, Kennedy Institute of Rheumatology, University of Oxford, Roosevelt Drive, Headington, Oxford, OX3 7FY, UK | Contributed to concept and design of study; interpretation of the data; critical review of the manuscript | Employee of Simomics |
| Mark Coles | The Kennedy Institute of Rheumatology, University of Oxford, Oxford, UK | Contributed to concept and design of study; interpretation of the data; critical review of the manuscript | None |
| Mark Curran | Janssen Research & Development | Contributed to concept and design of study; interpretation of the data; critical review of the manuscript | Employee of Janssen |
| Martin Hodge | Inflammation and Immunology RU, Worldwide Research & Development Pfizer Inc. | Contributed to concept and design of study; interpretation of the data; critical review of the manuscript | Employee of Pfizer |
| Martin Jenkins | Global Medicines Development, Astrazeneca | Contributed to concept and design of study; interpretation of the data; critical review of the manuscript | Employee of Astrazeneca |
| Mateusz Maciejewski | Inflammation and Immunology RU, Worldwide Research & Development Pfizer Inc. | Contributed to concept and design of study; interpretation of the data; critical review of the manuscript | Employee of Pfizer |
| Matt Page | UCB Celltech, Slough, United Kingdom | Contributed to concept and design of study; interpretation of the data; critical review of the manuscript | Employee of UCB |
| Matthew A. Sleeman | Respiratory, Inflammation & Autoimmunity, MedImmune Ltd, Cambridge, UK. | Contributed to the study design; provision of data interpretation; critical review of the manuscript | M. Sleeman was a full time employee of MedImmune, a wholly owned subsidiary of Astrazeneca. |
| Matthew J. Loza | Janssen Research & Development | Contributed to concept and design of study; interpretation of the data; critical review of the manuscript | Employee of Janssen |
| Maya Buch | University of Manchester (Centre for Musculoskeletal Research, School of Biological Sciences, Faculty of Biology, Medicine and Health, University of Manchester | Contributed to concept and design of study; interpretation of the data; critical review of the manuscript | None |
| Meilien Ho (1960-2016) | Global Medicines Development, Astrazeneca | Contributed to concept and design of study; interpretation of the data; critical review of the manuscript | Employee of Astrazeneca |
| Michael Binks | R&D GlaxoSmithKline, Stevenage, United Kingdom | Contributed to concept and design of study; interpretation of the data; critical review of the manuscript | Employee of GSK |
| Michael F. McDermott | Leeds Institute of Rheumatic and Musculoskeletal Medicine, University of Leeds | Contributed to concept and design of study; interpretation of the data; critical review of the manuscript | None |
| Michael Macoritto | Abbvie Bioresearch Center Inc., Worcester, MA, USA | Contributed to concept and design of study; interpretation of the data; critical review of the manuscript | Employee of Abbvie Inc |
| Michael R Barnes | Centre for Translational Bioinformatics, William Harvey Research Institute, Faculty of Medicine and Dentistry, Queen Mary University of London , London , UK | Contributed to concept and design of study; interpretation of the data; critical review of the manuscript | None |
| Michael R Ehrenstein | Division of Medicine, UCL | Contributed to concept and design of study; interpretation of the data; critical review of the manuscript | None |
| Michele Bombardieri | Centre for Experimental Medicine and Rheumatology, William Harvey Research Institute, Faculty of Medicine and Dentistry, Queen Mary University of London, London, UK | Contributed to concept and design of study; interpretation of the data; critical review of the manuscript | None |
| Myles Lewis | Centre for Experimental Medicine and Rheumatology, William Harvey Research Institute, Faculty of Medicine and Dentistry, Queen Mary University of London, London, UK | Contributed to concept and design of study; interpretation of the data; critical review of the manuscript | None |
| Neil Gozzard | UCB Pharma | Provision of strategic input and patient level data; critical review of manuscript | None |
| Neil Payne | Roche Products Ltd. 6 Falcon Way, Shire Park, Welwyn Garden City, AL7 1TW. UK | Contributed to concept and design of study; interpretation of the data; critical review of the manuscript | Employee of Roche Products ltd at the time of the study |
| Neil Ward | Department of Neuroscience, Amgen, Inc, Thousand Oaks, CA 91320, USA | Contributed to concept and design of study; interpretation of the data; critical review of the manuscript | Employee of Amgen Inc |
| Nina Joseph | Roche Products Ltd. 6 Falcon Way, Shire Park, Welwyn Garden City, AL7 1TW. UK | Contributed to concept and design of study; interpretation of the data; critical review of the manuscript | Employee of Roche Products ltd |
| Paul Emery | Leeds Institute of Rheumatic and Musculoskeletal Medicine, University of Leeds | Contributed to concept and design of study; interpretation of the data; critical review of the manuscript | None |
| Peter C. Taylor | Norman Collisson Professor of Musculoskeletal Sciences  Head of Clinical Sciences  Botnar Research Centre Nuffield Department of Orthopaedics, Rheumatology and Musculoskeletal Sciences, University of Oxford  Botnar Research Centre, Windmill Road  Headington, Oxford, OX3 7LD. | Contribution to the study concept; critical revision of manuscript | None |
| Peter Schulz-Knappe | Protagen AG, 44227 Dortmund, Otto-Hahn Street 15, Germany | Contributed to concept and design of study; interpretation of the data; critical review of the manuscript | Employee of Protagen AG |
| Petra Budde | Protagen AG, 44227 Dortmund, Otto-Hahn Street 15, Germany | Contributed to concept and design of study; interpretation of the data; critical review of the manuscript | Employee of Protagen AG |
| Philip Jones | University of Birmingham and University Hospitals Birmingham NHS Trust and Sandwell and West Birmingham Hospitals NHS Trust | Contributed to concept and design of study; interpretation of the data; critical review of the manuscript | None |
| Philip Stocks | NIHR Newcastle Biomedical Research Centre in Ageing and Long-term Conditions, Institute of Cellular Medicine, Newcastle University and Musculoskeletal Unit, Newcastle upon Tyne Hospitals NHS Foundation Trust | Contributed to concept and design of study; interpretation of the data; critical review of the manuscript | None |
| Rachel Harry | NIHR Newcastle Biomedical Research Centre in Ageing and Long-term Conditions, Institute of Cellular Medicine, Newcastle University and Musculoskeletal Unit, Newcastle upon Tyne Hospitals NHS Foundation Trust | Contributed to concept and design of study; interpretation of the data; critical review of the manuscript | None |
| Rafael Henkin | Centre for Translational Bioinformatics, William Harvey Research Institute, Faculty of Medicine and Dentistry, Queen Mary University of London , London , UK | Contributed to concept and design of study; interpretation of the data; critical review of the manuscript | None |
| Ravi Rao | R&D GlaxoSmithKline, Stevenage, United Kingdom | Contributed to concept and design of study; interpretation of the data; critical review of the manuscript | Employee of GSK |
| Ray Harris | Eisai Limited European Knowledge Centre Mosquito Way Hatfield Herts | Provision of advisory statistical input throughout the project and review of the statistical methodology | An employee of Eisai Limited. |
| Rekha Parmar | Leeds Institute of Rheumatic and Musculoskeletal Medicine, University of Leeds | Contributed to concept and design of study; interpretation of the data; critical review of the manuscript | None |
| Ruth Toward | Academic Department of Rheumatology, Division of Immunology, Infection and Inflammatory Disease, Faculty of Life Sciences, King’s College London | Contributed to concept and design of study; interpretation of the data; critical review of the manuscript | None |
| Sally Hollis | Biometrics and Information Sciences, Global Medicines Development, AstraZeneca | Contributed to statistical analyses of data; critical review of the manuscript | None |
| Samana Schwank | Academic Department of Rheumatology, Division of Immunology, Infection and Inflammatory Disease, Faculty of Life Sciences, King’s College London | Contributed to concept and design of study; interpretation of the data; critical review of the manuscript | None |
| Samantha Lipsky | Abbvie Bioresearch Center Inc., Worcester, MA, USA | Contributed to concept and design of study; interpretation of the data; critical review of the manuscript | Employee of Abbvie Inc |
| Samiul Hasan | R&D GlaxoSmithKline, Stevenage, United Kingdom | Contributed to concept and design of study; interpretation of the data; critical review of the manuscript | Employee of GSK |
| Sandra Martins | University College London (Division of Medicine) | Contributed to concept and design of study; interpretation of the data; critical review of the manuscript | None |
| Sandra Ng | Centre for Translational Bioinformatics, William Harvey Research Institute, Faculty of Medicine and Dentistry, Queen Mary University of London , London , UK | Contributed to concept and design of study; interpretation of the data; critical review of the manuscript | None |
| Sarah Brockbank | Institute of Cellular Medicine, Newcastle University | RA-MAP project manager: Overall management and facilitate the process of data acquisition and analysis | None |
| Sarah Keidel | Abbvie Bioresearch Center Inc., Worcester, MA, USA | Contributed to concept and design of study; interpretation of the data; critical review of the manuscript | Employee of Abbvie Inc |
| Scott Jelinsky | Inflammation and Immunology RU, Worldwide Research & Development Pfizer Inc. | Contributed to concept and design of study; interpretation of the data; critical review of the manuscript | Employee of Pfizer |
| Sharmila Rana | Centre for Experimental Medicine and Rheumatology, William Harvey Research Institute, Faculty of Medicine and Dentistry, Queen Mary University of London, London, UK | Contributed to concept and design of study; interpretation of the data; critical review of the manuscript | None |
| Simon Read | Grunenthal GmbH, Zieglerstraße 6, 52078 Aachen, Germany | Contributed to concept and design of study; interpretation of the data; critical review of the manuscript | Employee of Grunenthal GmbH |
| Stephen Kelly | Centre for Experimental Medicine and Rheumatology, William Harvey Research Institute, Faculty of Medicine and Dentistry, Queen Mary University of London, London, UK | Contributed to concept and design of study; interpretation of the data; critical review of the manuscript | None |
| Stephen Wright | Roche Products Ltd. 6 Falcon Way, Shire Park, Welwyn Garden City, AL7 1TW. UK | Contributed to concept and design of study; interpretation of the data; critical review of the manuscript | Employee of Roche Products ltd |
| Steve P Young | University of Birmingham and University Hospitals Birmingham NHS Trust and Sandwell and West Birmingham Hospitals NHS Trust | Contributed to concept and design of study; interpretation of the data; critical review of the manuscript | None |
| Sukru Kaymakcalan | Abbvie Bioresearch Center Inc., Worcester, MA, USA | Contributed to concept and design of study; interpretation of the data; critical review of the manuscript | Employee of Abbvie Inc |
| Susan Talbot | Department of Neuroscience, Amgen, Inc, Thousand Oaks, CA 91320, USA | Contributed to concept and design of study; interpretation of the data; critical review of the manuscript | Employee of Amgen Inc |
| Suzanne MM Verstappen | University of Manchester (Centre for Musculoskeletal Research, School of Biological Sciences, Faculty of Biology, Medicine and Health, University of Manchester | Contributed to concept and design of study; interpretation of the data; critical review of the manuscript | None |
| Tomi Lazarov | Department of Immunobiology, Division of Immunology, Infection and Inflammatory Disease, Faculty of Life Sciences, King’s College London | Contributed to concept and design of study; interpretation of the data; critical review of the manuscript | None |
| Tony Sabin | Medical Sciences Biostatistics group at Amgen | Provision and analysis of patient level data; input to statistical methodology; Contribution to the study concept; critical revision of manuscript | Former Amgen employee |
| Valerie Ludbrook | R&D GlaxoSmithKline, Stevenage, United Kingdom | Contributed to concept and design of study; interpretation of the data; critical review of the manuscript | Employee of GSK |
| Vernon Farewell | MRC Biostatistics Unit, University of Cambridge, UK | Contributed to concept and design of study; interpretation of the data; critical review of the manuscript | None |
| Wayne Tsuji | Medical Sciences, Early Development , Amgen | Provision of strategic input and patient level data; critical review of data | Former Amgen employee; Amgen shareholder |
| Wing Wu | Academic Department of Rheumatology, Division of Immunology, Infection and Inflammatory Disease, Faculty of Life Sciences, King’s College London | Contributed to concept and design of study; interpretation of the data; critical review of the manuscript | None |
| Wivine Burny | R&D GlaxoSmithKline, Stevenage, United Kingdom | Contributed to concept and design of study; interpretation of the data; critical review of the manuscript | Employee of GSK |
| Yujie Zhong | MRC Biostatistics Unit, University of Cambridge, UK | Statistical analysis, interpretation and revising manuscript critically | None |
| Zheng Liu | Abbvie Bioresearch Center Inc., Worcester, MA, USA | Contributed to concept and design of study; interpretation of the data; critical review of the manuscript | Employee of Abbvie Inc |
| Zhilong Jia | Centre for Translational Bioinformatics, William Harvey Research Institute, Faculty of Medicine and Dentistry, Queen Mary University of London , London , UK | Contributed to concept and design of study; interpretation of the data; critical review of the manuscript | None |
